# Automated multi-sample acquisition and analysis using atomic force microscopy for biomedical applications

**DOI:** 10.1371/journal.pone.0213853

**Published:** 2019-03-15

**Authors:** Antoine Dujardin, Peter De Wolf, Frank Lafont, Vincent Dupres

**Affiliations:** 1 Cellular Microbiology and Physics of Infection Group–Lille Centre for Infection and Immunity, CNRS UMR8204, INSERM U1019, Lille University Hospital Centre, University of Lille, Institut Pasteur de Lille, Lille, France; 2 Bruker Nano Surfaces Division, Santa Barbara, California, United States of America; Brandeis University, UNITED STATES

## Abstract

In the last 20 years, atomic force microscopy (AFM) has emerged as a ubiquitous technique in biological research, allowing the analysis of biological samples under near-physiological conditions from single molecules to living cells. Despite its growing use, the low process throughput remains a major drawback. Here, we propose a solution validated on a device allowing a fully automated, multi-sample analysis. Our approach is mainly designed to study samples in fluid and biological cells. As a proof of concept, we demonstrate its feasibility applied to detect and scan both fixed and living bacteria before completion of data processing. The effect of two distinct treatments (i.e. gentamicin and heating) is then evidenced on physical parameters of fixed *Yersinia pseudotuberculosis* bacteria. The multi-sample analysis presented allows an increase in the number of scanned samples while limiting the user’s input. Importantly, cantilever cleaning and control steps are performed regularly–as part of the automated process–to ensure consistent scanning quality. We discuss how such an approach is paving the way to AFM developments in medical and clinical fields, in which statistical significance of results is a prerequisite.

## Introduction

Based on the Scanning Tunneling Microscope (STM) [1], the Atomic Force Microscope (AFM) has initially been developed to operate on non-conducting surfaces [2]. Thanks to multiple developments, it is commonly used for research in many fields, including solid-state physics, polymer chemistry, molecular biology and cell biology. It has notably achieved a leading position in the semiconductor industry for quality control.

The immobilization of samples remains a prerequisite for any AFM experiment but the analysis of biological specimens imposes additional constraints. Firstly, most of the time, the experiments have to be carried out in aqueous medium. Secondly, the large dimensions and softness of cells required numerous improvements with the development of the contact mode [3] followed by oscillating modes [4] for which use in liquid media remains challenging.

In contrast to the aforementioned imaging modes, force-indentation spectroscopy gives quantitative information on mechanical properties of the sample. In modes such as “Force Volume”, these spectra are acquired on a 2D array of positions and the local properties can be mapped alongside the topography [5]. For example, on eukaryotic cells, AFM could bring important insights allowing to discriminate between normal and cancerous cells [6,7]. Nevertheless, acquisition mode remains slow in most cases with few samples analyzed at the time. However, significant improvements have recently been proposed with the development of new multiparametric AFM modes such as Quantitative Imaging (QI) and PeakForce Quantitative Nanomechanical Mapping (PF-QNM) [8]. Combining the force control of Force Volume with the speed of a scanning mode, PeakForce Tapping technology (Bruker) allowed to raster-scan the sample while doing a continuous vertical sinusoidal movement, at up to 8 kHz [9]. The feedback used is the peak force of the interaction between the tip and the sample, which corresponds to the force at the maximum extension of the piezo and gives control of the stresses applied to the sample. This was made possible in part by the emergence of new kinds of soft cantilevers with a high resonant frequency for tapping in fluid and specific tip shapes that allows for instance imaging of microvilli [10]. Nanomechanical mapping, with PF-QNM, can be achieved at high-speed on a large range of samples [9], although care has to be taken when using it on live cells, and remains under continuous developments [11].

Another caveat results from comparing data from different laboratories that gives widely different absolute values of the elasticity, even for the same cell lines [12]. This problem is being increasingly understood, as the main source of error originates from the calibration of the system. The use of pre-calibrated cantilevers allows for removing the incertitude on the cantilever spring constant, but the error on the deflection sensitivity still plays a major role. We, and others groups, have addressed this problem with the introduction of the Standardized Nanomechanical AFM Procedure (SNAP) [13]. It involves calculating a correction factor on the deflection sensitivity from the force constant of a calibrated cantilever, by reversing the thermal tune method.

The last major drawback, for which no satisfying middle- or high-throughput solution exists as of today, resides in the time-consuming operation-intensive AFM process in contrast to approaches routinely applied in the Omics fields. In AFM, the presence of a qualified operator is almost continuously required to carry out a series of tasks: reaching an area of interest, engaging the system, defining the scan parameters, finding the cell, zooming on the elements of interest, fine-tuning the imaging parameters, saving the data, and moving to the next cell… On the other hand, because of the complexity of the cell, the between-group variance of measurements is often quite small compared to their within-group variance. As a consequence, experiments described in many publications are carried out with too few samples to ensure proper statistical significance of the results. Statistically-sound data are also important to detect small subpopulation of an isogenic sample, which can sometimes dominate macroscopic behavior [14]. A typical example is found in bacterial persistence to antibiotics [15] or through chronic or latent infections [16], where stochastic phenotypical changes can be of high importance.

To increase the throughput of the process, one approach is to enhance the number of scans per unit of time. This could be done by increasing the scanning speed to process more cells in a row or by parallelizing the system to scan several cells at the same time. Scanning speed increases are on their way, partially driven by development carried by High-Speed AFM [17], with improvements in the electronics and other components as well as in the technique as such [18,19]. Since the late 1990s, several parallelization systems using cantilever arrays have been developed, with up to millions of cantilevers operating in parallel [20–22]. These devices are, however, unsuitable to living biological samples. A similar system has been devised, using interferometry, to operate on fixed fibroblasts [23]. The multiple tips are aligned on a fixed grid and at the same height whereas the cells vary in height and position. Hence, each cantilever will probe different parts of the cells with very different forces. This adds variability to the resulting data and may damage the sample. Groups of miniaturized AFMs [24] have also been introduced, but they do not seem to be adapted to biological conditions since they operate in air, on an inverted sample, and their electronics are not water compatible.

Automation of the process is another alternative that has the advantage of being less dependent on operator availability. Many systems already implement some degrees of automation. For instance, the Dimension FastScan AFM has the ability to realign the photodetector before engaging automatically. The possibility to automate the scanning process as such depends on the mode being used. Although tapping mode is very useful for some purposes such as for fast topography imaging, it can be rather difficult to operate in fluid. In contact mode, the vertical deflection is subject to drift in such proportions that it can quickly become larger than the setpoint. For this reason, we did not attempt in automation imaging recording. An automated setpoint adjustment exists [25], although it has not been shown on live cells. In PeakForce Tapping, the ScanAsyst technology is available and allows the user to define a fixed force setpoint or let the algorithm optimize the parameters. Some fully automated AFM instruments exist, with primary applications in quality-assessment of semiconductor wafers. Among them, only a few systems are able to host multiple samples. However, they are only able to scan in air, which prevents any large application to biological systems. As a consequence, we propose in this study a solution that consists in automating the procedure on a sequential but multi-sample basis operating in fluid applied to biological cells. Although the proofs of concept shown in this work focus on prokaryotes, most elements should be transferable to eukaryotic cells opening avenues to the use in middle and high throughput biomedical applications.

## Material and methods

### Experimental set-up

**Fig 1:**
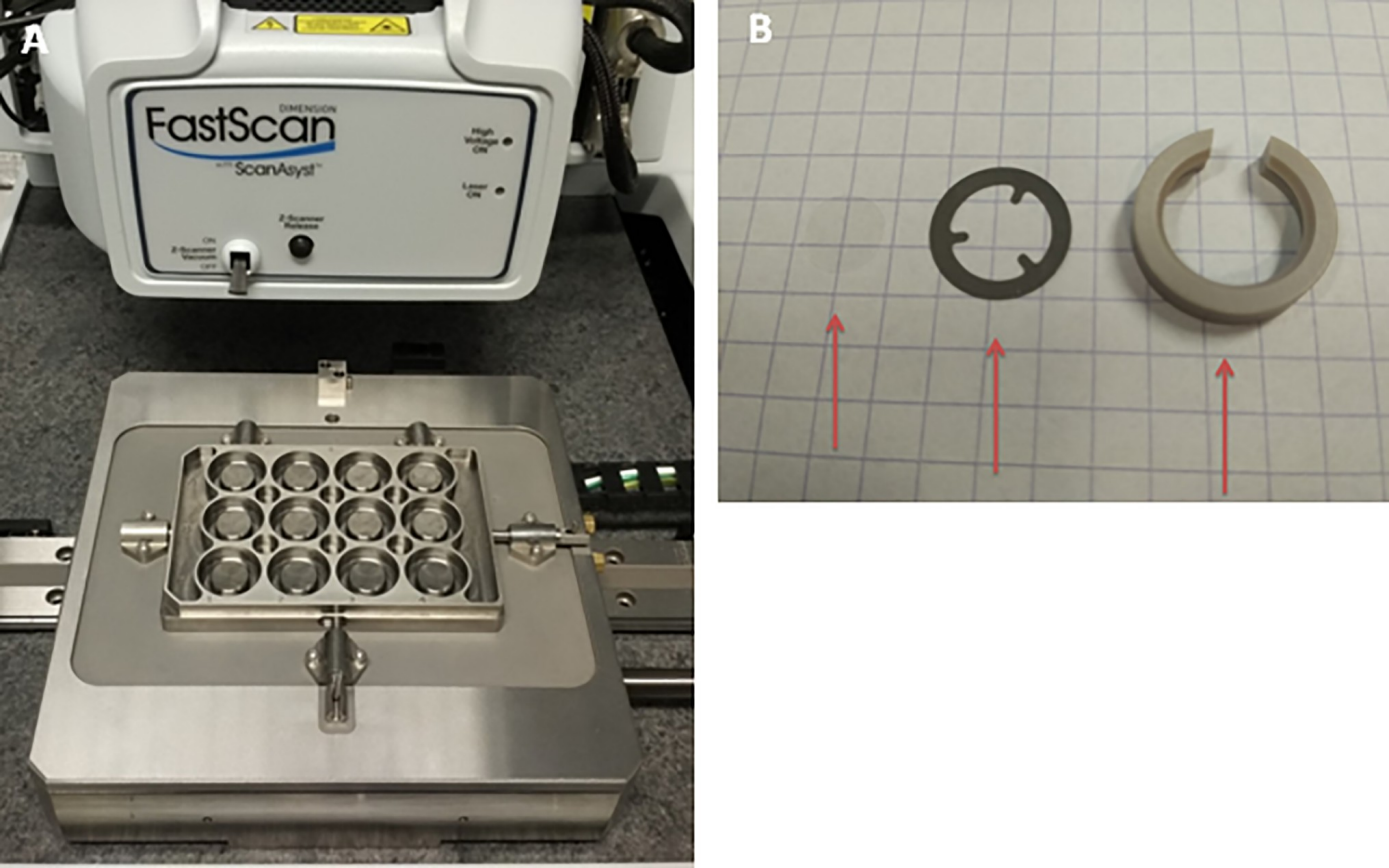
Experimental setup. (A) Picture of the Multi-Well plate and its attachment to the Dimension FastScan-Bio AFM. (B) From left to right: round 8 mm coverslip, spring used to hold the coverslip, spring clamp used to secure the spring and the coverslip to the Multi-Well plate. The grid is 5 mm by 5 mm.

The system is based on a Dimension FastScan-Bio AFM (Bruker, Santa Barbara, CA, USA). The tip is mounted on a z-scanner that can be used in fluid. The stage of the FastScan-Bio AFM has been modified to host a sample holder able to hold a sample in each of its 12 wells (3 × 4). This Multi-Well plate can be removed and replaced with accuracy such that the vertical position is known to within a few hundred nanometers. The modified stage with the Multi-Well plate is shown in Fig 1A. Each of the wells can hold a small round coverslip with a diameter of 8 mm, in fluid (Menzel-Gläser, Braunschweig, DE). The coverslip is secured with a bio-compatible spring and a spring clamp, as shown in Fig 1B.

### Software

The Dimension FastScan-Bio AFM is controlled by the Nanoscope 9.3 software. A Python interpreter was integrated, providing high-level scripting capabilities. Access to the Nanoscope functionalities is emulated as a Python package available within the interpreter. It gives read and write accesses to the necessary parameters such as the scan rate and scan size. It also ensures reading access to the common measurements such as the current value of the photodetector sum and the deflection. It is, furthermore, able to control the stage, save the optical image, engage, start a scan, capture SPM files, and withdraw. From these general-purpose capabilities, a specialized Python package has been created to give a higher-level interface to the requirements of the experiment, whose functionalities are described below.

### Bacterial sample preparation

The samples used for the present experiment were fixed *Yersinia pseudotuberculosis* and living *Mycobacterium bovis* BCG bacteria.

*Yersinia pseudotuberculosis* (IP32777 wild-type strain) cultures were prepared overnight in LB broth at 28°C in an agitated incubator. They were then diluted at 1% the next morning in 3 Falcon tubes (25 mL) and cultured. After 4h, when the solution was turbid (OD_600_ exponential phase), the 3 cultures were centrifuged at 3000 × g for 5 minutes. For one third of the sample, the medium was replaced by fresh medium and kept at ambient temperature. For the second third, the new medium was replaced by medium containing 8 μg/mL gentamicin and kept at ambient temperature. For the last third, the medium was replaced by fresh medium and kept at 60°C in a water bath. All media were preheated.

In the meantime, 8 mm round coverslips were placed in 3 Multidish 4 Well (NUNC, Roskilde, Denmark) and cleaned by Oxygen Plasma at 50 W for 5 minutes. After 1 h, 400 mL of each culture were put in each well of the corresponding Multidish. All Multidishes were then centrifuged at 3000 × g for 5 minutes. Each sample was washed 3 times with potassium phosphate buffer saline (PBS), fixed during 20 minutes with formalin solution (10% neutral buffered–Sigma Aldrich), and washed 3 times with PBS. Tris buffer was then used for 5 minutes to quench formalin reactivity. The samples were then washed one last time with PBS before being thoroughly rinsed with distilled water and dried.

Before the experiment, one sample of each Multidish was rehydrated for 20 minutes in 1 mL PBS so that it could be removed from its well before being mounted as described below.

*Mycobacterium bovis* BCG was grown in Sauton medium as described elsewhere [26]. As these bacteria also tend to form aggregates, 0.15 mL of the cells suspension (OD_600_ 2.3) was sonicated for 1 min and very slightly centrifuged for a few seconds. A small volume (0.1 mL) of the supernatant was diluted at 10% in PBS. Then, 8 mm round coverslips were placed in a Multidish 4 Well (NUNC, Roskilde, Denmark) and cleaned by Oxygen Plasma at 50 W for 5 minutes. 0.4 mL of the solution was put in a well and centrifuged at 2000 × g for 5 minutes. The coverslip was then mounted as described below.

Each sample was then placed on a well of our custom Multi-Well and was secured with the spring and spring clamp. An additional clean coverslip was placed and secured as well in a fourth well for being used for calibration purposes. Wells with a coverslip were rinsed 3 times with PBS. The Multi-Well plate was then placed and secured on the stage of the FastScan AFM and PBS was added to fill the wells. An empty well was filled with 70% ethanol solution, which can be used for cleaning purposes.

### Description of the experiment

#### Nanoscope parameters

A Nanoscope experiment was previously defined from the profile of the default experiment for PeakForce Tapping in Fluid. This default profile was modified to accommodate a Height Engage of 500 nm with a 2 s delay. Force Volume parameters were set to 64 by 64 pixels and 30 μm by 30 μm for the scan size, 10 Hz for the ramp rate, and 500 nm for the ramp size. PeakForce parameters were set to 128 by 128 pixels for the scan size, 5 nN for the ScanAsyst SetPoint (ScanAsyst autocontrol was disabled for the SetPoint but kept for the gains and others), 5 nm for the ScanAsyst noise threshold, 3 Hz for the scan rate, 1 kHz for the PeakForce Frequency, and 300 nm for the PeakForce Amplitude. The experiment was then saved for further uses.

#### Start-up

Nanoscope was started with the previously defined experiment parameters. The user, only required during the setup of the experiment, loads the probe (ScanAsyst-Fluid–Bruker, Santa Barbara, CA, USA) and does the setup and calibration in the dedicated well. The setup consists of the selection of the probe parameters, the selection of the tip location, and the alignment of the laser and photodetector, using the standard interface of the software. For the calibration, we currently use the standard touch calibration, which consists of calculating the deflection sensitivity and spring constant using force curves and thermal tune. For future experiments, calibrated tips and SNAP could be used [13]. The PeakForce parameters, the sync distance and the PeakForce Sensitivity, were also measured at the selected PeakForce Frequency (here 1 kHz). The Force Volume trigger threshold was then set to 1 nm. The user finally defines the target size, which will be used as the mask size to detect bacteria as well as for the size of the measurement scan. For these experiments, the size was chosen to be 2 μm squared. Once the setup is done, the user can launch the script of the experiment and is no longer required.

#### Well changing

Given the high accuracy of the Multi-Well plate and its attachment to the AFM stage, the centers of the samples are at fixed and known positions. Given the flatness of the system, the height can be measured once and recorded, with a constant sample clearance. Moving to a well is done by moving the head up to a secure height. The stage moves horizontally to the desired well. The head is then lowered up to the sample position plus some clearance, with the optics focusing on the sample.

Since this movement implies going from air to fluid, bubbles may appear between the glass and the cantilever. The probability for this to happen is, however, very dependent on the type of cantilever and the cleanliness of the system. After entering each well, the laser sum is checked to be close enough to the original one in order to detect big defects. An image of the cantilever is then saved so that the user can check for the absence of bubbles *a posteriori*. Using ScanAsyst-Fluid probes and by working cleanly, the appearance of bubbles is, from our experience, quite exceptional.

To remove the bubbles (or tip contamination), the system is moved in a well filled with a solution of ethanol and water. The cantilever is then rinsed in the calibration well before going to the sample.

#### Engaging

The maximum range of the Dimension FastScan-Bio AFM in the horizontal dimensions is 30 μm by 30 μm. To cover a larger area, a survey scan is realized every 30 μm over an array (here 3 by 3) of positions. For each well, the system is engaged sequentially at each position.

One of the most critical steps in AFM is the alignment of the laser and the photodetector, prior to starting the engaging process. In most AFMs, these two alignments have to be done manually but some microscopes are motorized and can be aligned from the controller. The user is also supposed to bring the sample and the tip within the range of the piezo, although the movement can also be made by motors. Automated methods of alignment and engagement have been developed quite early on [27]. In these systems, the laser position is set by the user during the setup phase and appears to be quite stable. The Nanoscope software is, by default, able to realign the photodetector and perform the engagement, here in PeakForce mode.

The script then switches to Force Volume mode and a low-resolution (here 64 pixels squared) Force Volume is captured and used as a survey scan. Although we are only interested in imaging the sample, Force Volume was chosen on this large but low-resolution scan because its speed depends mostly on the resolution (1 curve per pixel) whereas imaging modes are mostly limited by the scan size, since they need to track the sample during raster-scanning. Raster-scanning at high-speed on large distance can also be damaging for the sample.

#### Detection

The survey scan is analyzed automatically. In the cases of obviously aberrant scans, i.e. when sudden steps or an abnormally large number of “hits” are detected, the position is dropped and the scan moves on to the next survey area. Such aberrance might be due to contaminants or defects in the area or to contamination of the cantilever during scanning.

Otherwise, the data is flattened at the first order to remove the tilt. A height threshold is defined, below the expected size of bacteria, here at 200 nm higher than the background. Iteratively, the local maximum of the flattened image is taken, as long as it stands higher than the threshold. A square mask of the size of the target (defined earlier, here 2 μm squared) is centered on this point. The mask is repositioned on the center of mass of its intersection with the data above the threshold. This location is stored and the area falling under the mask is excluded for future calculation. The procedure is then reiterated until all data falls below the threshold.

#### Scan

Once the positions have been detected, the script switches the system back into scan mode, in PeakForce-QNM, although we did not use the QNM channels in the analysis. The scan size is set to the size of the mask (here 2 μm) and the scan is sequentially offset to the determined positions. Additional time is then given for ScanAsyst to optimize the scan parameters and a capture is taken for each local sample.

The size of the scan (and the corresponding mask) is set to the target size, which was chosen here to be 2 μm squared to have a relatively high resolution of the texture of the bacteria, although a smaller value could have been chosen to have a better resolution. Should the bacteria be well-separated on the sample, 4 μm squared scans can be used to scan entire and isolated bacteria, although with a lower resolution.

### Analysis

After the experiment, the scans were quickly visually checked on Nanoscope Analysis to remove those showing no bacteria or of which the bacteria part was clearly negligible. Further checks were added in the analysis to ignore files for which the bacteria occupied less than 5% of the usable area of the scan. The remaining scans were deemed of “good quality”. Having a quick manual filtering is a step that we consider important to guarantee the soundness of the data and that takes negligible time. This filtering aims at removing obvious artifacts and empty scans based on the “qualitative” experience of the user, while the automated filtering removes border-line cases based on quantitative criteria. In the case of a perfectly characterized system whose quality is guaranteed, the manual filtering could be dropped to lead to a fully-automated process. This implies being able to afford a few percents of potentially corrupted data in the analysis. Most of the filtered-out files would likely be removed by the analysis, but a few might still be analyzed and yield outliers in the data, which can still be visually checked post-analysis.

In order to illustrate the interest of gathering such an important number of scans, we observed a few parameters on each sample using the aforementioned Python library. For each scan, the height image was smoothed by a 2D Gaussian filter with sigma = 3 pixels (1 pixel = 15.625 nm in our conditions). The background level was defined as the minimum of the smoothed image. The area of interest was defined as everything that was 300 nm higher than the background and for which the error signal was higher than -5 nN (the SetPoint value). Otherwise, it would indicate a poor tracking of the sample and can be seen to happen on the glass. This mask was eroded by 10 pixels so as to limit the area of interest to a flatter area of the cell and get rid of artifacts happening on the glass. Every element smaller than 100 pixels was discarded from the scan, as well as every scan for which less than 5% of the usable part of the scan (i.e. after removal of 10 pixels on the border due to the erosion) was kept at that stage.

#### Height

The height of each sample was measured as the difference between the maximum and minimum values of the height on the smoothed image.

#### Roughness

The roughness of the sample was also measured at the scale of interest, as the arithmetical mean deviation from the smoothed data. However, a common problem of AFM images is the slight variation from one line to another due to the raster-scanning, which creates an artificial roughness along the slow axis. As a consequence, for this measurement, the lines along the fast axis were smoothed individually in order to determine the profile. This measurement represents the roughness of elements on the order of 30 nm or smaller.

#### Statistical significance

Student’s t-test can be used to compare distributions by contrasting their means. One assumption is that the errors on the mean are equal to each other (in theory), or at least can be approximated as such. This is the case for (but not only) distributions with the same number of samples and the same variations. When comparing distributions with different numbers of samples, the error on the mean can be different for each distribution. In this case, the simple t-test has to be generalized by weighting the distributions proportionately, which is known as Welch’s t-test [28].

## Results and discussion

**Fig 2:**
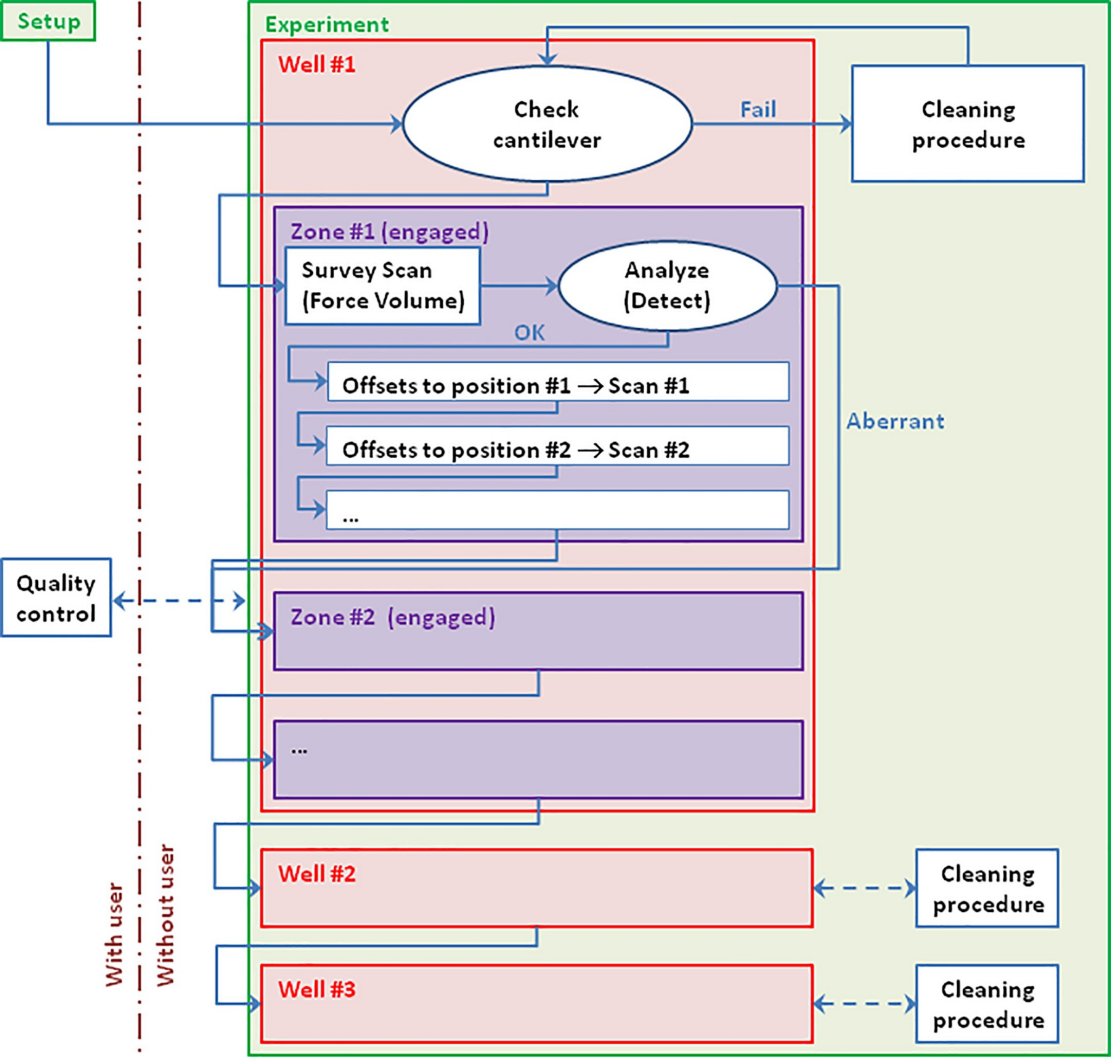
Schema detailing the workflow of bacteria detection and scanning. Once the setup has been establisheed by the user, the experiment can be started. During the experiment, the system goes through the different wells and checks the cantilever upon each entry. A cleaning procedure (outside of the well) is triggered if necessary. In a well, it engages in the different zones, in which it takes a survey scan to detect the bacteria, which are then scanned with subsequent high-resolution scans.

A Dimension FastScan-Bio AFM was used with a custom Multi-Well (shown in Fig 1) able to hold 12 samples on small coverslips. The Nanoscope software controlling the system has been modified to integrate a Python interpreter, which allows to design an automated experiment workflow, as illustrated in Fig 2.

After an initial setup by the user in a well dedicated to the calibration, the experiment can be started and the system goes into the first well of interest (well #1). Since moving between wells implies crossing back and forth the liquid-air interface, this can be prone to defects, such as air bubbles on top of the cantilever. The absence of contaminant on the cantilever is asserted by automatically checking the soundness of the laser signal while an image of the tip is saved for subsequent analysis. Otherwise, a cleaning procedure is triggered, and reiterated if necessary. The sample is then divided in several areas, each one having the range size of the AFM piezos. The stage moves within the well to the first zone and the system engages the tip on the sample. After engaging, a low-resolution Force Volume survey scan is taken over the full available extent and analyzed to detect cells (bacteria in this case). The system then switches scan mode to PeakForce Tapping with ScanAsyst and performs detailed scans on the detected positions. Once these scans have been performed, or in the case where the analysis has concluded in a corrupted capture (which might be due to a tip contamination, contamination on the sample, or other artifacts), the system withdraws and performs the same procedure on the other zones of interest on the sample. Once the target zones of a sample have all been scanned, the system moves to well #2 (and higher) and follows the same instructions (including the cleaning procedure).

### Multi-cells

**Fig 3:**
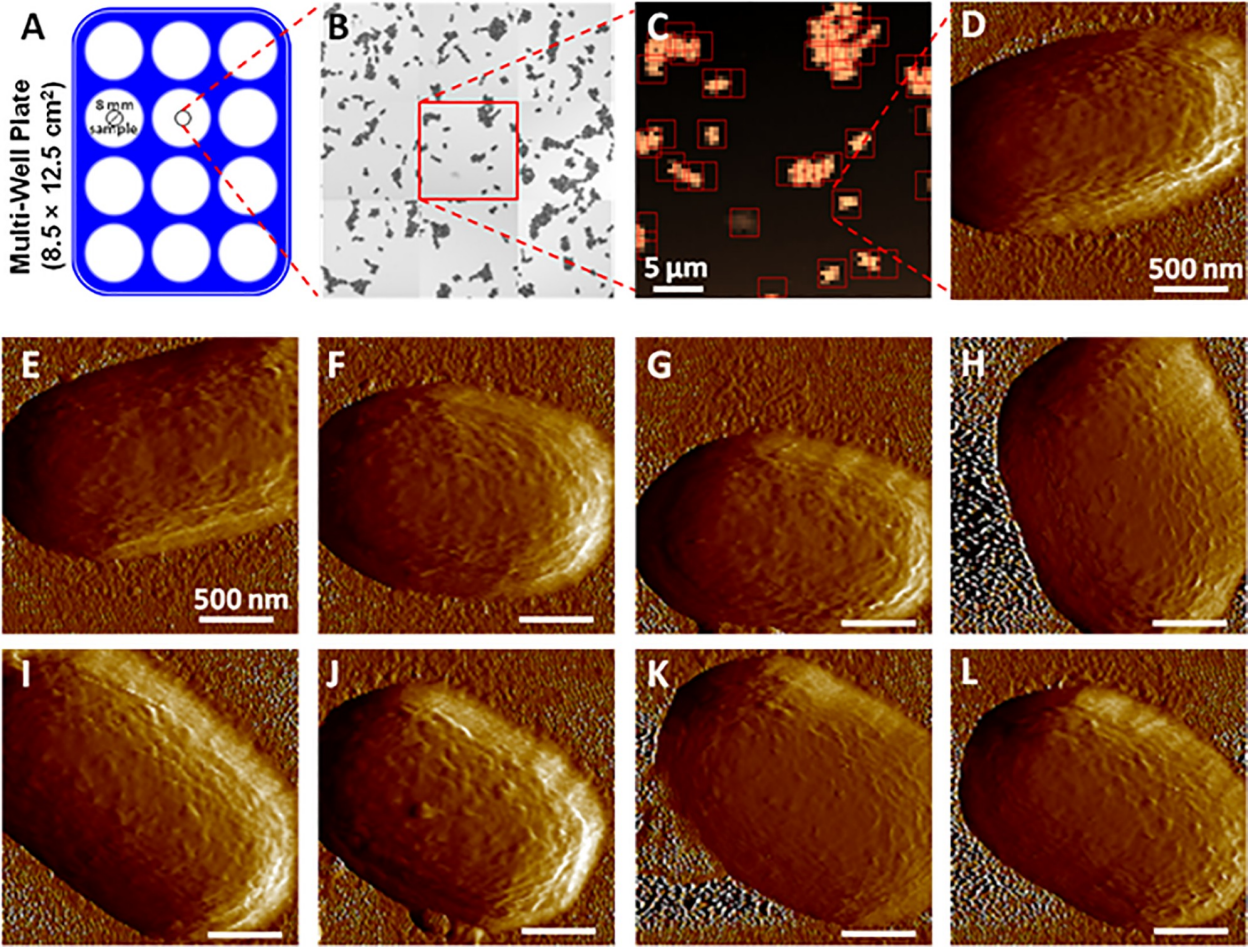
Illustration of the workflow of *Yersinia pseudotuberculosis* bacteria detection and scanning. (A) Schematic representation of the Multi-Well plate. (B) Image reconstructed from the 3-by-3 array of Force Volume capture survey scans taken in the control well. (C) One of the Force Volume height maps from [B], with detected areas of interest squared as detected automatically during the experiment (image generated automatically during the scanning process). (D) Peak Force Error map of one of the bacteria in [C], taken automatically. (E-L) Peak Force Error maps of other bacteria, taken from different subparts of [B].

As a proof of concept, we used a sample of fixed *Y*. *pseudotuberculosis* bacteria. After sample preparation, bacteria appear randomly distributed on the glass surface (Fig 3B). The whole detection and scanning process is illustrated in Fig 3. One can notice that after an initial setup by the user, the experiment was started and the presence of the user was no longer required for data acquisition.

The bacteria were first detected with a Force Volume survey scan but, given the maximal extension of 30 μm of the piezos in both horizontal directions, covering a wider area required reiterating survey scans on an array of positions. Here, a total area of 90 μm by 90 μm was chosen for the survey in the center of the well, which was implemented by a 3 by 3 array of 30 μm by 30 μm Force Volume images, represented in Fig 3B. Each Force Volume image (Fig 3C) was analyzed in near-realtime (just after the capture), to identify 2 μm by 2 μm areas of interest, represented as squares on Fig 3C. All these areas of interest were then automatically scanned in PeakForce Tapping. Fig 3D shows the Peak Force Error image of a bacteria detected in Fig 3C. Fig 3E to 3L provide other examples of bacteria images from other parts of the surveyed area.

From the whole set of Force Volume files analyzed during this experiment, a total of 501 areas of interest were detected in 8 h 35 min. Among them, 16 were removed because i) other objects than bacteria have been detected (for instance bacterial fragments or debris that can be generated during sample preparation) or ii) the bacteria occupied less than 5% of the recorded scan. Representative images obtained in such cases are shown in S1 Fig. The remaining scans, i.e. 485 scans, were considered of quality suitable for analysis, which corresponds to a good-quality scan being produced every 64 seconds on average. This clearly highlights the effectiveness of the system in terms of the number of samples automatically detected and analyzed.

The duration of the entire process can be broken down as follows: both the engaging process and the survey scan in Force Volume took between 8 and 9 minutes, most of which is taken by the Force Volume (about 7 minutes for 64 × 64 curves at 10 Hz). Detecting the bacteria and switching to PeakForce mode took about 40 seconds before the selected areas could be scanned at a rate of one every 50 seconds. Experiments were performed several times with similar results in terms of bacteria detection, provided that the sample preparation was successful. One can notice that the process can be fully customized according to the number of cells to be detected and the resolution required for each scan.

The Multi-Well prototype in this study can be further optimized for wider use for instance by producing sterile plastic-made culture dishes. Alternatively, promising methods include dedicated microfluidics systems allowing to distribute bacteria on arrays with properly designed cubicle [29] or micropatterned adherent substrata onto which bacteria can adhere [30] or mammalian cells can be seeded [31,32].

### Multi-Sample

**Fig 4:**
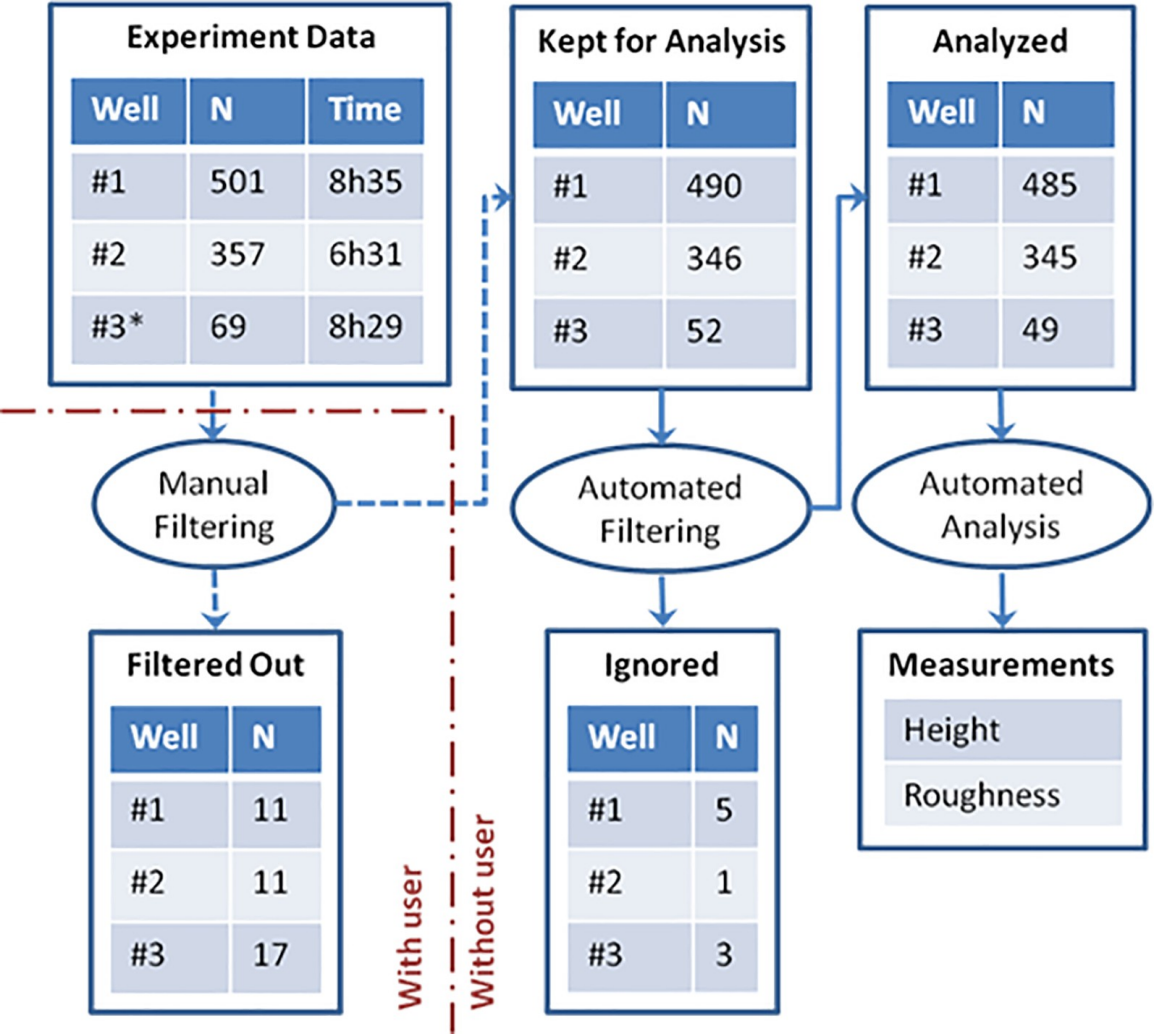
Schema of the analysis. The experiment data for the different wells and the corresponding total time of scanning is shown on the top-left corner. The data is first checked visually for obvious artifacts. A second filter is part of the analysis routine. For each of the analyzed scans, a measure of height and roughness is extracted.

Next, vs. control sample of *Y*. *pseudotuberculosis* bacteria (#1, Fig 4), we scanned samples treated with gentamicin (#2, Fig 4), an antibiotic used for the treatment of serious infections due to Gram-negative bacteria and bacteria heated at 60°C for 60 minutes (#3, Fig 4). All samples were simultaneously and identically prepared on coverslips, fixed, and mounted to the Multi-Well plate, to be scanned in fluid on the system. Due to the limited number of bacteria in sample #3, a total area of 210 μm by 210 μm was scanned to yield a satisfactory amount of data, instead of the 90 μm by 90 μm one. As illustrated in Fig 4, a total of 357 scans were gathered for sample #2 and 69 for sample #3, among which 345 and 49 were kept for analysis. Otherwise, a target number of cells could have been set by the user to define a number of scans per conditions.

**Fig 5:**
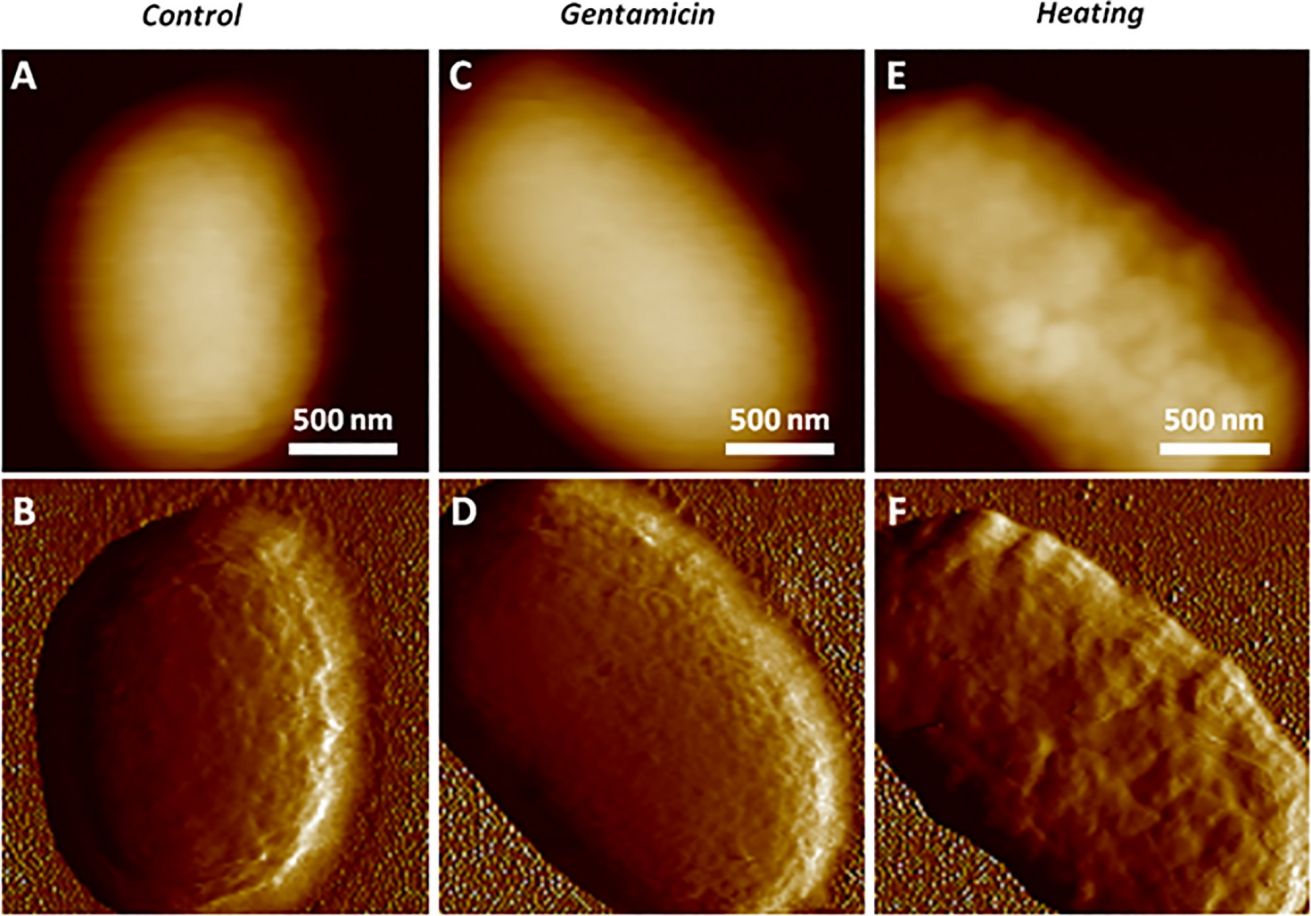
Height (A-C-E) and Peak Force Error (B-D-F) AFM images of representative bacteria from the control sample (#1, A-B), the gentamicin-treated sample (#2, C-D), and from the heat-treated sample (#3, E-F).

For each sample, a representative height map recorded at a position of interest and the corresponding PeakForce error image are shown in Fig 5. Both height and PeakForce error images were simultaneously acquired. Although the height image contains the actual height data, it appears blurred due to the large curvature of the bacteria. The error signal has, however, an intrinsic lightning/shadowing effect coming from the slopes of the sample, which gives a visual clue of the topography and reveals fine surface details. Nevertheless, it should be kept in mind that PeakForce error images do not provide quantitative height measurements and the height images are the only ones that can be used for analysis, in particular for all height and roughness measurements. Big lumps can be observed at the surface of the control bacteria (Fig 5B) whereas the gentamicin-treated ones seem to have lost them and present very fine ripples (Fig 5D). On the other hand, the heat-treated sample shows strong evidence of a significantly altered membrane (Fig 5F). Although the third condition is clearly different from the two other ones, the difference between the first two cases is more subtle and it remains difficult to rule out that such a difference could be a visual artifact or due to random variations in the populations.

**Fig 6:**
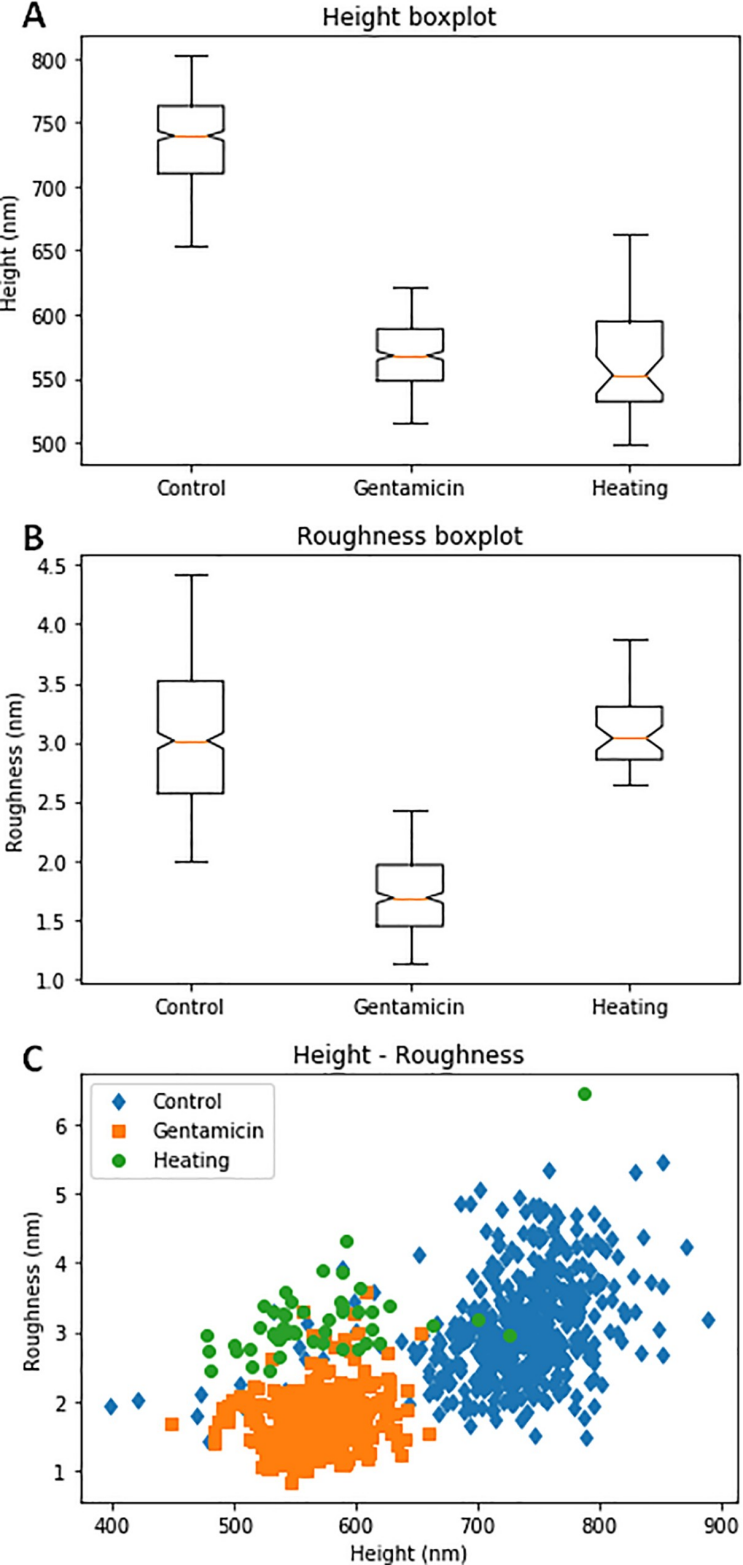
Representations of the height and roughness distributions of the control (#1), gentamicin-treated (#2), and heating-treated (#3) samples. (A) Boxplot representation of the height data. Welch's t-test p-values: 1.8252e-260 (Control–Gentamicin), 0.9028 (Gentamicin–Heating), 1.3440e-24 (Heating–Control). (B) Boxplot representation of the roughness data. Welch's t-test p-values: 1.7503e-154 (Control–Gentamicin), 5.9971e-22 (Gentamicin–Heating), 0.3281 (Heating–Control). (C) Scatter-plot of the roughness vs. height data.

To quantify these differences, we extracted height and roughness on all the gathered data for the three conditions in an automated manner. As shown in Fig 6A, a reduction in height of about 25% is observed between the control and both the gentamicin- and heat-treated cases. The gentamicin treatment shows a significant alteration of the surface through a significantly reduced roughness (Fig 6B), which could confirm the visual observation of the disappearance of the lumps on the surface. The heat-treated sample shows a different alteration of the sample.

Thus, the height enables the control to be differentiated from the other two conditions. On the other hand, the roughness allows for the differentiation of the gentamicin-treated sample. Height and roughness could then be used to create a 2D scatterplot (Fig 6C). This plot clearly illustrates the clustering of the three conditions over these two parameters.

A Welch’s t-test was used to highlight the significance of the results (values in Fig 6). The differences between the control and the gentamicin-treated sample, although visually subtle, clearly appear in the numbers with an overwhelmingly high significance (p-value < 10^−150^). Because of the comparatively low number of samples in the last condition, the p-values related to that condition are much higher than the ones related to the difference between the control and gentamicin treatment but are still smaller than 10^−20^ for the significant differences.

In our case, a main asset of the system was the ability to scan the three samples with the same tip and without user intervention prone to corrupting the calibration (e.g. changing the alignment of the laser). As long as the samples were stable for the time of the analysis, which is not a problem for fixed samples, we can consider the three conditions to be equivalent except for the treatments, to which the observed differences can be attributed.

It is also worth noting that since all the data was saved, the evolution of the parameters over time can easily be analyzed. It has been done during the experiment on *Y*. *pseudotuberculosis* to ensure that cell morphology (through height and roughness) was not altered during the process (S2 Fig). This kind of control can be particularly useful in experiments on living cells in which a temporal evolution of cell morphology must be ruled out.

### Living cells

Based on the previous results with fixed bacteria, we were interested in verifying that our approach could also be used on living bacteria. As a model, we chose living *Mycobacterium bovis* bacillus Calmette Guérin (BCG). Due to the hydrophobic nature of their surface, *M*. *bovis* BCG cells adhere spontaneously to surfaces [33].

**Fig 7:**
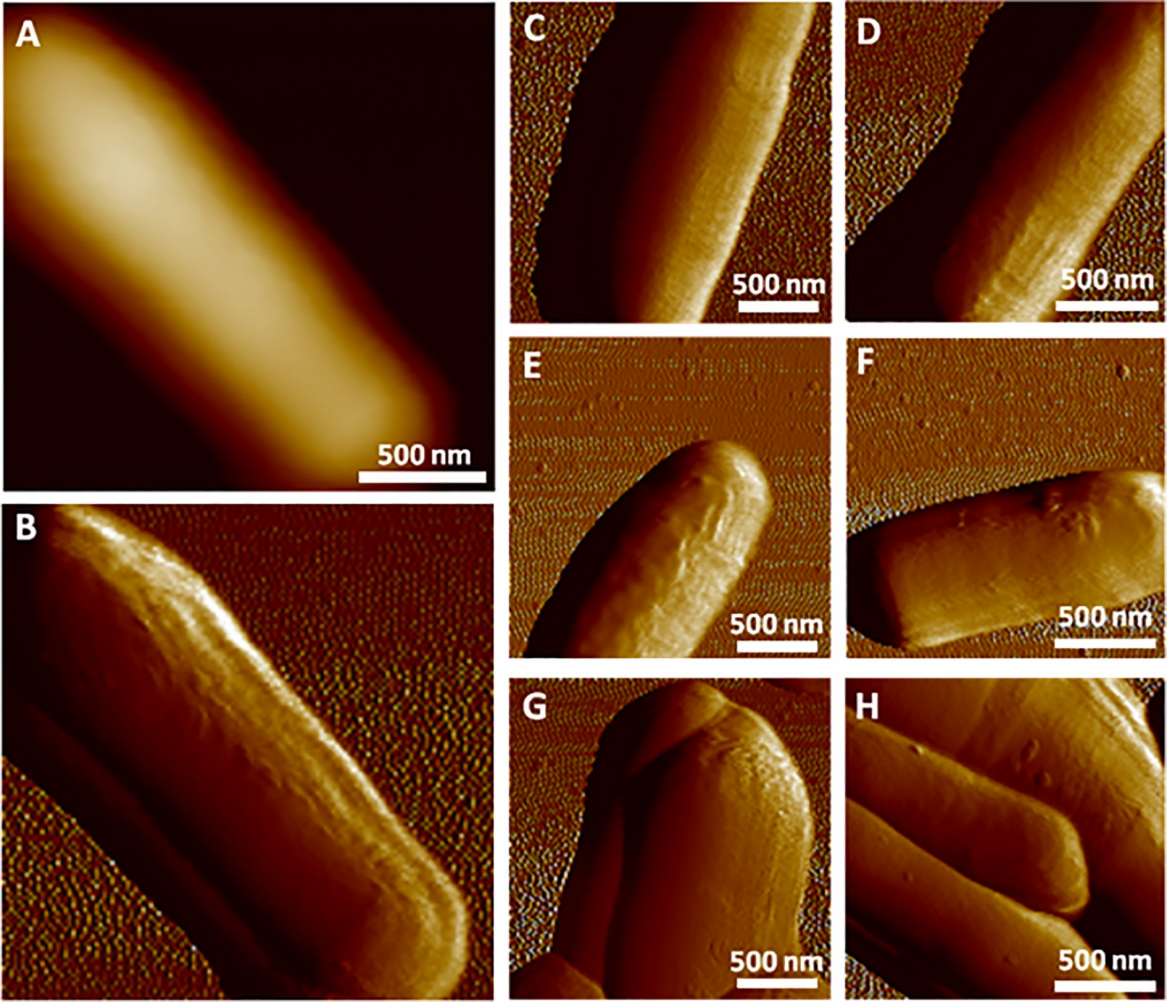
AFM imaging of living *M*. *bovis* BCG cells. (A) Height and (B) PeakForce error images of an isolated *M*. *bovis* BCG cell. (C-H) PeakForce error images of several living mycobacteria detected.

As shown in Fig 7, *M*. *bovis* BCG bacteria were successfully detected and scanned on the sample. We have noticed the presence of both isolated bacteria (Fig 7A–7F) and aggregates (Fig 7G and 7H). Topographic images of the cell surface could be recorded without significant modification of the surface morphology and reveal a smooth and homogeneous surface, which is consistent with previous AFM observations [34,35].

*M*. *bovis* BCG cells have a characteristic length ranging from 2 to 5 μm while *Y*. *pseudotuberculosis* bacteria length is typically around 1.5 μm. Given the size difference, it would have been possible to take a bigger mask size to scan an entire BCG cell at once. We chose, however, to use exactly the same parameters to illustrate the robustness of the system (i.e. that the cells could be correctly detected even with non-optimized parameters). Obviously, the scan parameters can be adapted according to the desired application, i.e. for instance, large scale to quantify cell parameters or smaller ones for low-scale imaging (for instance 500 nm × 500 nm on the cell to perform roughness measurements).

The mask used for cell detection was a square. However, it would be possible to define a more relevant scan shape for the bacteria, such as a rectangular one aligning itself with the bacteria. Taking 2 μm scans on BCG is useful to gain an insight of the roughness and the height of the bacteria, but one could be more interested in the roughness of smaller scan areas. In this case, it would be relevant to refine the detection with another low-resolution scan instead of the measurement scan, after which smaller and more accurate scans could be carried out. These precise roughness measurements on various positions of different bacteria and obtained for several sample conditions could have a great interest in, for instance, antibiotic development. Once the tools made available, possibilities are almost limitless, although quite dependent on the application in sight. In this study, we have performed high-resolution topographical measurements. This requires the use of an imaging mode and only PeakForce Tapping appears to us to be compatible with such a level of automation in liquid. But it is worth noting that nanomechanical properties could be recorded with other modes as well, such as Force Volume, QI, PF-QNM or any other ramp-based methods. Force Volume and PF-QNM are already available through our device and other ramp-based modes can easily be implemented during further developments.

Thus, BCG cells were analyzed using PF-QNM mode even if we did not use the QNM channels since we focused on topographical images. Nevertheless, PF-QNM mode allows to obtain biophysical parameters (elasticity, adhesion, rupture forces…) and could hence be acquired automatically during the process. Typical adhesion and elasticity maps of the surface of BCG cell are shown in S3 Fig.

A crucial point to consider when scanning (prokaryotic and eukaryotic) cells is that biomolecules in the culture medium and small dirt or bacteria can sometimes contaminate the cantilever and interfere with the laser path. A user will normally stop the experiment and change or clean the cantilever, before going back to the sample. Moving from a sample to another one causes the crossing of air-solution interfaces bringing another challenge: the tendency for bubbles to form. All of these make the corresponding automation quite difficult, in that a constant quality control is needed to ensure that the experiment is progressing error-free. It is comparatively more challenging than the current levels of automation on quality control in air, where i) the sample is generally much better controlled, ii) less unexpected events can happen and iii) basic checks and a tip characterization are usually sufficient. Although such problems have never been encountered during the experiments covered by this work, we are fully aware of the need to take this eventuality into account. Thus, some solutions have already been implemented at the time of these experiments. Contaminations and bubbles in the path of the laser can be detected by controlling the photo-detector sum signal. Bubbles at other places of the cantilever can be detected by image processing. We attempted to get rid of them by moving to a well filled with an ethanol solution, followed by a rinse in the calibration well before going back to the sample. Contaminations hopping on or off the tip can be detected in the Force Volume map by looking for steps in average height of a scan line. A more refined tip control system is being developed, in which a tip check sample is used in liquid to control the quality of the tip and attempt to free the tip from contaminations. Speed and reactivity are being improved as well, with a goal to reach industry-level reliability and turn-key full automation.

## Conclusion

We are convinced that AFM has a huge potential in the biomedical field and, more broadly, in healthcare-related research at different scales. This technique remains, however, highly demanding since data collection on large numbers of cells is needed. It implies a lot of time and efforts from its operators, which can cause researchers to sometimes draw conclusions from observations based on too little data. To cope with this, a good solution is to gather enough data to ensure statistical significance. Also, a lot of effort has been done and is still being done in making AFM faster, but achieving high-speed introduces a parameter such as viscosity that has to be taken into account and for which theoretical models in this context have to be validated [36].

A procedure and a variety of scripts were developed to make the instrument able to automatically move from one sample to another and scan autonomously hundreds of fixed *Yersinia pseudotuberculosis* bacteria in liquid in a few hours, with data analysis performed in the flow which was previously unheard of. A statistical analysis was then performed on the obtained results to highlight the relevance of the increase in the number of samples. It should be noted that the tool is flexible in that the scripts can be adjusted to each sample type. The proofs of concept are demonstrated on fixed and living prokaryotes but most elements should be transferable to eukaryotic cells. However, further developments are necessary since such cells require, in particular, precise temperature control.

Automation could be used on a larger number of conditions with fewer samples per condition in order to increase the throughput of AFM sample-wise. It could then operate as a high-information screening that may also help to improve pre-clinical screening in drug discoveries [37]. With AFM as a medium-throughput scanning on top of its high-information capabilities, it could be integrated to the drug discovery pipeline to lower the attrition rate at the final stages of the development cycles of new drugs and then reduce the costs and cycle times of pharmaceutical R&D [38], which is facing huge challenges with regards to the current low output of drug discoveries [39–41].

## Supporting information

S1 Fig Peak Force Error AFM images of scans discarded during analysis. (PDF)

S2 Fig Time evolution of height and roughness measurements for control and gentamicin-treated samples. (PDF)

S3 Fig FD-based AFM topography (A) and directly correlated multiparametric maps (B-D) of a living *M*. *bovis* BCG cell.
(PDF)
