# Supplementary figures and images for: Automated multi-sample acquisition and analysis using atomic force microscopy for biomedical applications
Source: PLoS One. 2019 Mar 15;14(3):e0213853. doi: 10.1371/journal.pone.0213853 (PMC6420161; doi:10.1371/journal.pone.0213853)

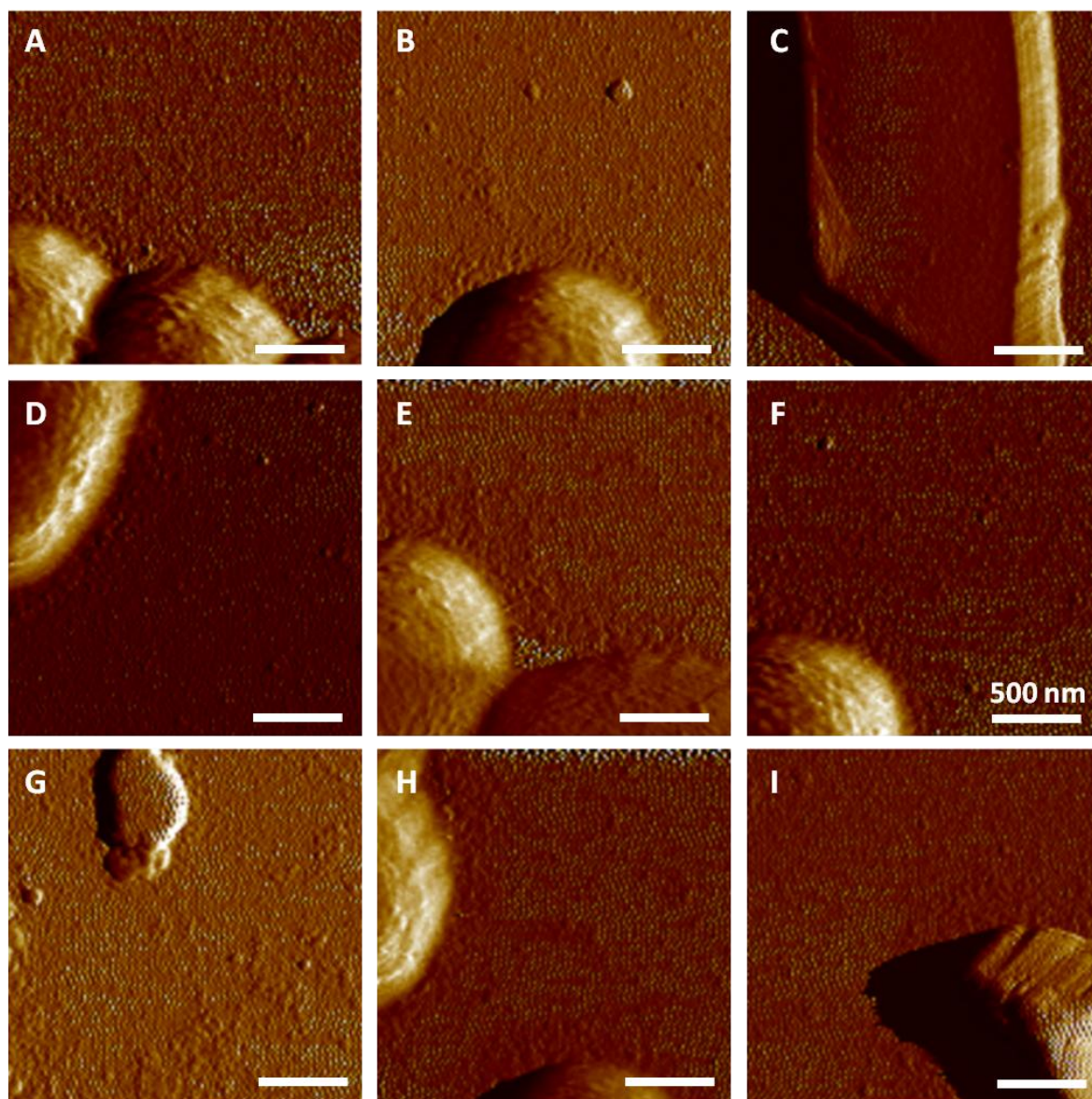

Figure S1

Supplement: S1 Fig — (PDF) [file pone.0213853.s001.pdf]

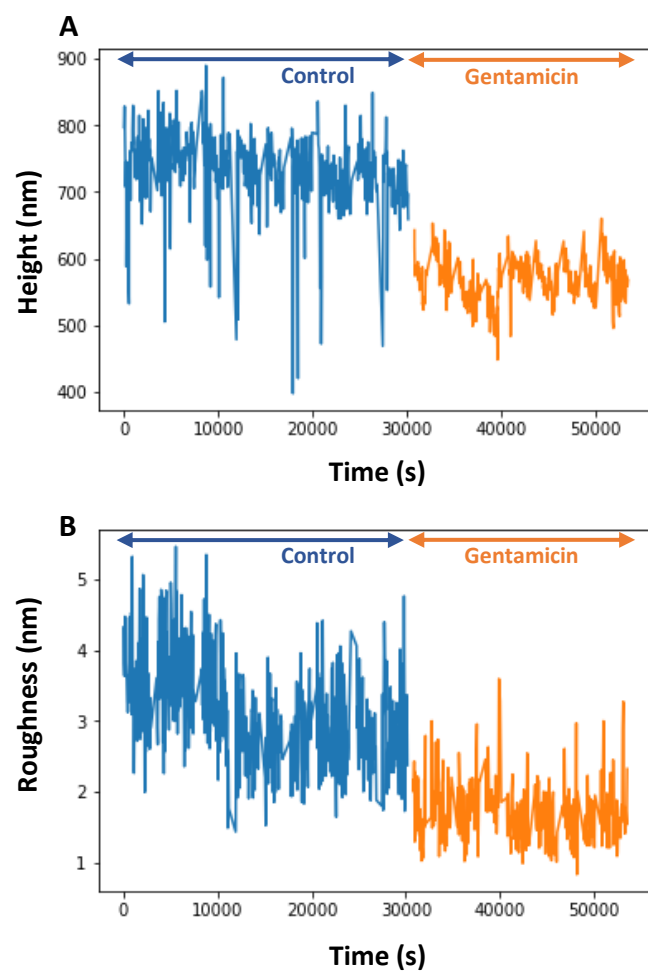

Figure S2

Supplement: S2 Fig — (PDF) [file pone.0213853.s002.pdf]

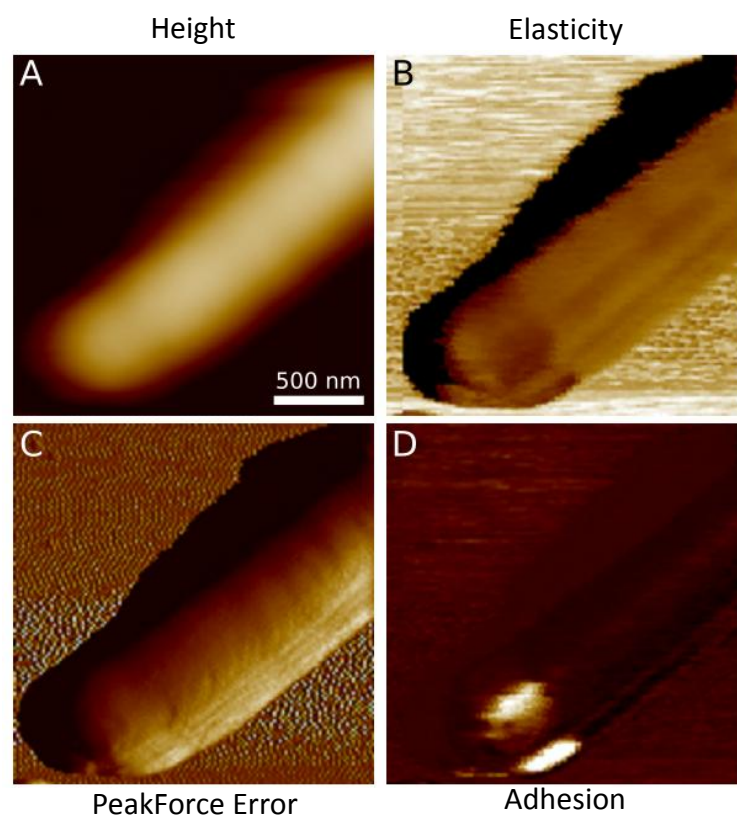

Figure S3

Supplement: S3 Fig — FD-based AFM topography (A) and directly correlated multiparametric maps (B-D) of a living M. bovis BCG cell. (PDF) [file pone.0213853.s003.pdf]
